# Artificial intelligence-powered personalized patient dosimetry in CT

**DOI:** 10.1093/bjrai/ubaf007

**Published:** 2025-05-08

**Authors:** John Damilakis

**Affiliations:** Department of Medical Physics, School of Medicine, University of Crete, 71003 Iraklion, Crete, Greece

**Keywords:** X-ray imaging, machine learning, artificial intelligence, deep learning, deep neural networks, convolutional neural networks, radiation, dosimetry

## Abstract

Advanced X-ray imaging techniques have increased the importance of patient dosimetry, requiring better dose assessment methods and improved dose management strategies to optimize imaging protocols, resulting in diagnostic information obtained with the lowest possible dose to the patient. This review explores the potential of artificial intelligence (AI) in CT dosimetry. Using machine learning models, trained with extensive data from personalized Monte Carlo dosimetry, AI significantly enhances the dosimetry process by providing accurate, rapid dose assessments. It automates organ segmentation from CT images, a critical and traditionally time-consuming step, enabling precise estimation of organ doses. AI’s ability to predict dose distributions with high accuracy facilitates the seamless integration of personalized dosimetry into clinical workflows, promising improved patient safety and optimized radiation dose management. Additionally, AI-driven approaches offer substantial advancements over conventional methodologies, which often face challenges such as prolonged computation times and labour-intensive manual processes. Despite these advancements, challenges hinder the widespread adoption of AI in CT dosimetry. These include potential biases in training data and the need for robust validation to ensure accuracy across diverse patient populations and imaging conditions. Addressing these challenges is essential for realizing the full potential of AI-powered dosimetry in clinical practice.

## Introduction

As X-ray imaging advances with more complex and higher-dose procedures, the importance of patient dosimetry increases. Despite advancements in dose-reduction techniques, procedures such as CT often involve high radiation exposure. Patient dosimetry enables personalized dose optimization, real-time monitoring, and compliance with safety standards. Research and innovation in this field are essential to refine dose assessment methods, improve dose management strategies, and foster the development of new technologies designed to reduce radiation exposure based on the specific clinical scenario, patient size, and anatomical area of interest.^1^

Artificial intelligence (AI) is a rapidly evolving field in X-ray imaging.^2^^,^^3^ By using machine learning (ML) models trained on a wealth of ground truth data provided by personalized Monte Carlo (MC) dosimetry, AI has the capability to revolutionize this field.^4^ It can significantly expedite the dosimetry process, providing accurate dose assessments in a fraction of the time currently required. AI algorithms can automate the segmentation of organs from CT images,^5^ bypassing one of the most time-consuming steps in the MC dosimetry workflow. This is very important for personalized patient dosimetry where organ segmentation is a necessary step to estimate organ doses. Furthermore, AI can predict dose distributions with high accuracy, thus facilitating rapid, personalized dosimetry that could be seamlessly integrated into clinical workflows.^6^

By enabling real-time, patient-specific dose estimations, AI enhances radiation dose management. This improvement directly contributes to patient safety by reducing unnecessary radiation exposure and ensuring adherence to the as low as reasonably achievable principle. Additionally, AI-driven dosimetry allows for wider clinical implementation, making personalized dosimetry more accessible across healthcare settings, including those with limited resources. These advancements pave the way for integrating personalized dosimetry into routine diagnostic radiology, supporting both improved radiation protection and enhanced imaging practices.

The aim of this review is to provide information about the present state of AI-driven patient dosimetry in CT, including its prospects and challenges.

## Existing methods for patient dose estimation

Patient dosimetry in X-ray imaging has advanced significantly with the integration of methods such as optically stimulated luminescence dosimetry, thermoluminescence dosimetry (TLD), MC simulation algorithms, and voxelized or mesh-based phantoms. TLD, a well-established technique, measures doses using materials that emit light when heated after radiation exposure. Its energy response and linearity make it effective for various applications in X-ray medical imaging. TLDs are often combined with physical anthropomorphic phantoms, which simulate human anatomy and tissue composition for accurate dose measurements.

MC simulation algorithms have transformed patient dosimetry by enabling highly accurate, particle-level dose estimations in complex geometries. These methods excel in modelling specific patient anatomy using mathematical phantoms. The goal for radiation dose assessment in CT is personalized MC dosimetry, as it provides highly accurate and patient-specific estimations. This method has been verified^7^^,^^8^ and utilizes sophisticated simulation algorithms and models constructed from each patient’s CT images, thereby enabling the determination of dose distribution within a patient’s body. Patient models enable the representation of individual patient anatomy in MC simulations, thereby facilitating personalized dosimetry assessments.

Despite the accuracy of MC dosimetry, it is marred by lengthy computation times, which can vary significantly depending on the complexity of the simulation and the computational resources available. Moreover, the manual segmentation of organs for extracting organ/tissue-specific dose values remains a labour-intensive bottleneck, impeding the widespread adoption of personalized dosimetry in diagnostic radiology.

## Importance of rapid dose prediction

Rapid patient dose estimation is essential in X-ray imaging. Although performed post-acquisition, fast dose calculations allow for radiation exposure analysis for every patient. This enables adjustments for future imaging procedures and supports AI-driven decision-making in radiation dose management. Patient organ dose estimation in X-ray imaging using conventional methodologies is a complex and time-consuming process, which has been a significant barrier to its integration into clinical practice. Traditional methods such as MC simulation experiments require detailed modelling of the X-ray machine’s geometry, the patient’s anatomy, and the precise conditions of the imaging procedure, remaining essential for generating initial data to train AI algorithms, which are critical for future AI inferences. This often involves intricate calculations and simulations that can take considerable time to complete, which remain crucial in the data generation phase for accurate modelling despite advancements in AI. Additionally, the variability in patient size, shape, and tissue composition adds layers of complexity, necessitating personalized adjustments to ensure accuracy.^9^ Another source of uncertainty, besides organ segmentation, is the variability in organ composition. Although AI can greatly assist with organ segmentation, addressing differences in tissue composition remains a challenge that must be resolved for more precise patient-specific dose estimations. Due to these complexities, real-time or rapid organ dose estimation has been challenging to achieve. As a result, despite the clear benefits of organ-specific dose information for optimizing patient safety and image quality, such detailed dosimetry has not become commonplace in everyday clinical settings. An AI-powered automated patient dose estimation process has the potential to revolutionize this approach by providing accurate, real-time dose assessments.

## The machine learning workflow

The use of ML in X-ray imaging is a rapidly growing field, offering the potential to enhance diagnostic accuracy, speed, and efficiency.^10^ The ML workflow in this context involves several critical steps, each contributing to the development of reliable and effective models. An overview of the process is provided below.

### Acquisition of data

The first step is acquiring X-ray images, dose values and other data to be used for training and testing the ML model. These data can come from various sources, including radiographs, mammograms, fluoroscopy and interventional procedures and CT scans. The data typically include imaging data (eg, pixel data from DICOM files) and metadata extracted from the DICOM headers, such as patient demographics, imaging parameters (eg, tube current, exposure time, and kVp), and study information. It is crucial to ensure that the dataset is representative of the diverse range of cases the model will encounter in real-world applications. This includes variations in patient demographics, disease stages, and imaging equipment.

### Data preprocessing

Preprocessing involves preparing the data for analysis, which may include data cleaning, noise reduction, and normalization. In some cases, data augmentation and resizing are also necessary. Data augmentation refers to techniques used to artificially increase the size and diversity of the training dataset by applying transformations to the existing data. Common examples include rotating, flipping, scaling, cropping, or applying random noise to images. For instance, in medical imaging, augmenting data by flipping or rotating an X-ray can help the model generalize better to variations in patient positioning. The goal of these preprocessing steps is to improve the quality of the input data and make it more suitable for ML models.

### Feature engineering

This step involves identifying and extracting relevant features that the model will use to make predictions. In traditional ML approaches, domain expertise is vital for designing effective features. However, deep learning models, particularly convolutional neural networks (CNNs), can automatically learn complex features directly from the image data.

### Model selection

This step is related to the selection of an appropriate ML model or architecture based on the problem, data characteristics, and computational resources. This could range from simple models like logistic regression to complex neural networks. The model selection depends on several factors, including the nature of the task (classification, segmentation, etc.), the size and quality of the dataset, and the computational efficiency required.

### Model training and testing

Model training involves using a subset of the data (training set) to teach the model to make predictions. This process can follow different paradigms, such as supervised learning, where labelled data is used to train the model; unsupervised learning, which identifies patterns or clusters in unlabelled data; or semi-supervised learning, which combines labelled and unlabelled data. Testing, on the other hand, evaluates the model’s performance on a separate set of data (test set) that it has not seen before. It is essential to use a robust validation strategy, for example, cross-validation to ensure the model generalizes well to new data and to prevent overfitting.

### Model evaluation

This step assesses the model’s performance using appropriate metrics tailored to the specific task. For classification tasks, metrics such as accuracy, precision, recall, F1 score, and the area under the receiver operating characteristic curve are commonly used. For regression tasks, evaluation metrics may include mean absolute error, mean squared error, root mean squared error, and the coefficient of determination (*R*^2^). The choice of metrics should reflect the clinical relevance and the specific objectives of the task.

### External validation

External validation involves testing the model on a completely independent dataset, often from different institutions, to evaluate its generalizability and robustness in real-world settings. Successful external validation is crucial for demonstrating the model’s potential clinical utility across diverse patient populations and imaging protocols.

### Model deployment

Model deployment refers to the process of making a trained ML model available for use in a production environment. This involves several considerations to ensure that the model performs well in real-world scenarios and can be accessed by end users. Deployment challenges include integrating the model with existing healthcare information technology (IT) systems, ensuring compliance with regulatory requirements, and establishing protocols for model maintenance and updates.

### Support

The final step involves the practical implementation of the model within the operational environment. The model starts to interact with live data and provides outputs that can be used for decision-making. Continuous monitoring and maintenance are crucial to ensure the model remains accurate and relevant. This step involves updating the model with new data and refining it as needed. Providing support ensures that any issues with the model are promptly addressed and that users can effectively use the model. This includes technical support and user training.

**Figure 1.**
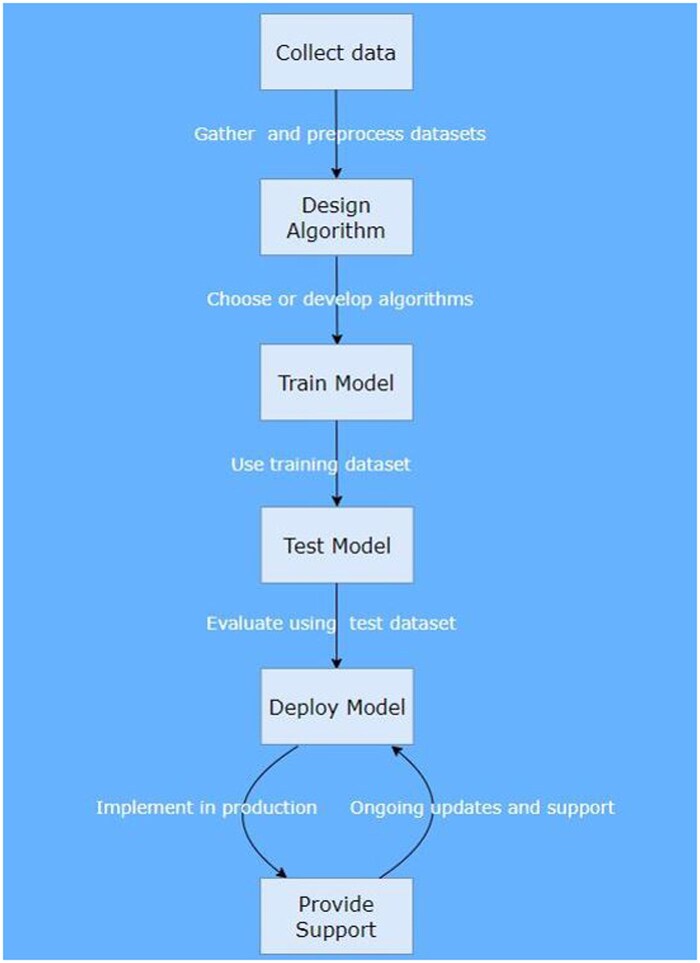
A typical workflow for developing and deploying a machine learning model for patient dosimetry in X-ray imaging.

Figure 1 illustrates the basic steps of a typical workflow for developing and deploying an ML model for patient dosimetry in X-ray imaging. Throughout this workflow, ethical considerations, data privacy, and regulatory compliance are paramount, especially given the sensitive nature of medical data. Additionally, continuous monitoring and updating of the model are essential to maintain its performance and relevance over time.

Several frameworks are widely used for developing AI models in medical imaging and related fields. Popular deep learning libraries such as PyTorch and TensorFlow provide robust tools for building and training neural networks, offering flexibility and scalability for diverse applications. Monai, a specialized framework built on PyTorch, is tailored for medical imaging tasks, providing pre-built components for segmentation, registration, and classification. Additionally, scikit-learn remains a versatile library for implementing classical ML algorithms and preprocessing techniques. These frameworks have become integral to advancing AI-driven solutions in medical research and practice.

## Emerging AI models and their potential application in patient dosimetry

Recent advancements in AI have introduced new architectures and methodologies that hold significant promises for patient dosimetry. These models offer unique strengths and address challenges posed by traditional approaches.

### Transformers

Initially developed for natural language processing, Transformers have recently been adapted for computer vision tasks, demonstrating state-of-the-art performance in image analysis. Their ability to capture long-range dependencies and global context in data makes them a potential candidate for tasks like dose estimation, where complex spatial relationships between anatomical structures and radiation fields are critical. Transformers could be leveraged for direct mapping of 3D dose distributions from imaging data, offering scalability and improved accuracy.

### Recurrent neural networks

Recurrent neural networks are well suited for sequential data, making them a viable option for modelling attenuation-based dose distributions in CT scans, where the order of slice acquisition plays a role. They can also be used in combination with convolutional layers to model temporal variations or patient motion during imaging procedures.

### Transfer learning models

Transfer learning has proven highly effective in domains with limited datasets, such as patient-specific dosimetry. Pretrained models, fine-tuned on medical imaging datasets, can leverage knowledge from larger datasets, accelerating training and improving performance in dose prediction tasks. This approach is particularly useful when dealing with specific modalities or rare cases where data scarcity is a concern.

### Ensemble models

Ensemble models combine predictions from multiple algorithms, increasing robustness and accuracy. For example, combining deep learning networks trained on imaging data with ML models using patient demographics and DICOM metadata could enhance dose estimation by integrating diverse information sources. This approach mitigates the risk of overfitting and improves generalizability across varied patient populations.

### Attenuation-based models

These models focus on modelling the interaction between X-rays and tissues using physical principles, often integrating AI techniques for enhanced accuracy. By incorporating patient-specific anatomical data and imaging parameters, such models can provide highly accurate dose estimations tailored to individual patients.

### Pros and cons of different models

While newer models like Transformers offer improved accuracy and scalability, their computational demands can be significant, requiring substantial hardware resources for training and deployment. Modern ensemble methods, such as hybrid models combining CNNs with traditional ML algorithms, or ensembles of deep learning architectures trained on diverse datasets, have the potential to mitigate individual model biases, enhance generalizability across different patient populations, and improve dose estimation precision. Simpler architectures like CNNs or traditional regression models are computationally efficient but may lack the ability to model complex relationships in the data. The choice of model should consider trade-offs between accuracy, computational efficiency, and ease of implementation.

## Studies using AI for the prediction of patient dose

**Figure 2.**
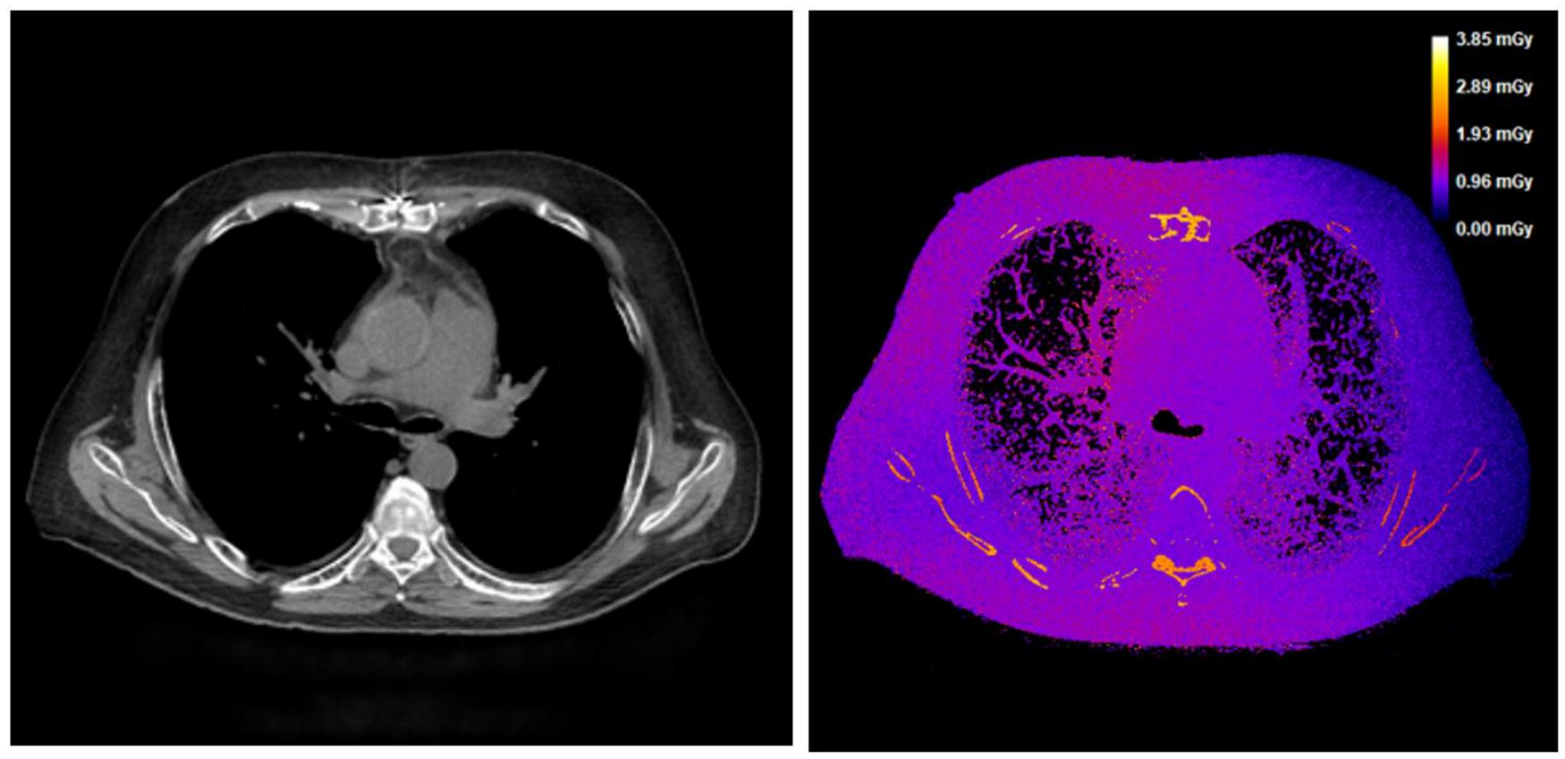
Chest CT scan (left) and corresponding dose image (right) depicting radiation dose distribution.

Several recent studies have been published with the aim of replacing traditional MC dose simulations. Many of these studies employed scanner-specific and patient-specific MC simulations to calculate organ doses, using these as a ground truth. The output from the MC software is presented as a three-dimensional dose distribution (Figure 2).

Tzanis and Damilakis presented an ML-based methodology for predicting patient-specific organ/tissue doses associated with head CT examinations.^11^ By analysing CT data from 343 patients, they developed a deep neural network (DNN) model trained with data from 231 head CT examinations. This model predicts doses to the brain, eye lenses, and cranial bones with minimal discrepancy compared to MC simulations, demonstrating accuracy and a significant reduction in computational time (97%). A user-friendly software tool was also developed to facilitate dose/risk assessment in clinical practice. This methodology offers a promising shift towards accurate, patient-specific radiation dose estimation and risk assessment, highlighting its potential applicability in enhancing CT dosimetry.

A fully automated ML-based methodology for personalized radiation dose assessment has been developed to improve the accuracy and efficiency of radiation dose assessments in CT scans of the thoracic and abdominal regions.^12^ The researchers utilized 331 thoracoabdominal radiotherapy-planning CT examinations for training and validation of 3D U-Nets for organ segmentation. U-Nets are a type of CNN architecture designed for pixel-level predictions, where each pixel in an image can be assigned to a specific category in classification tasks or a continuous value in regression analyses. Additionally, diagnostic thoracic and abdominal CT exams were collected to develop and validate a DNN for organ dose prediction. The effectiveness of these AI models was tested against the gold-standard MC simulations. The study demonstrated that the ML models could predict radiation doses with a mean percentage difference of under 8% compared to MC simulations across various organs. This precision was achieved with a significant reduction in processing time, that is, 99% faster than the MC methods.

A rapid, DL-based method has been developed for estimating patient-specific organ doses from thoracic CT examinations.^13^ Utilizing CT data from 95 individuals, MC simulations were performed to create 3D dose distributions for heart, lungs, oesophagus, and bones. A deep learning network was trained with variables such as water-equivalent diameter (WED), scan length, and tube current to predict organ doses. The method, tested on an independent dataset of 19 patients, demonstrated very good agreement with MC simulations, with an average difference of −5.1%, 4.3%, 0.9%, and 1.4% for the heart, oesophagus, lungs, and bones, respectively. This approach simplifies organ dose estimation, potentially enhancing patient care by providing immediate dose assessments without the need for extensive image processing or segmentation.

The researchers of a recent study^14^ employed a DNN to predict MC dose maps based on CT scans. The model used whole-body CT scans from 63 patients, with various acquisition parameters, including tube voltages and current modulation, to train the network. They utilized a residual DNN architecture for image regression to predict the absorbed dose for individual patients. The model was tested on 11 whole-body CT scans, evaluating its accuracy using several metrics, such as mean error, mean absolute error, relative error, and relative absolute error. The voxel-wise performance for 120 kVp with tube current modulation showed a high correlation between the DL-generated dose maps and the MC-generated dose maps, indicating that the model was able to replicate the results with a relatively low error rate. The average relative error for all segmented organs was below 4.5%.

Juszczyk et al present an analysis and application of a DL-based system for estimating the size-specific dose estimates (SSDE) in CT.^15^ The research demonstrates the use of a CNN for the semantic segmentation of the body area in axial CT slices, which is central to determining the patient’s effective diameter and the WED. The researchers compared several modes of SSDE determination against results provided by a commercial system. The comparison was done across different body regions like the head, chest, and abdomen. The results suggest some notable insights, particularly in the head region, where the study recommends double-checking the localizer-based SSDE methods.

A computational approach, referred to as deep dose estimation (DDE), has been introduced, which leverages CNNs to estimate patient-specific radiation doses during CT scans.^16^ The DDE system predicts dose distributions using a U-Net-like architecture, which inputs CT images and first-order dose estimates. The model’s validation involved several technical setups, including different scan trajectories, voltages, and anatomical regions, using both training data and an independent set of CT scans from the Visual Concept Extraction Challenge in Radiology (Visceral) project. The results demonstrated that DDE could generalize well across various scanning parameters and anatomical sites, showing a maximum mean absolute percentage error of 6.3% and minimum gamma passing rates of 91%, which are competitive with real MC simulations. This study also evaluated the DDE’s capability in predicting organ-specific doses. The DDE system showed high accuracy, with average errors around 3.1% for organ doses, validating its clinical utility. Furthermore, the DDE operates efficiently, processing a whole-body CT scan in approximately 1.5 s, offering a feasible alternative to traditional MC methods.

A very recent study introduces a method for predicting radiation doses to specific brain organs during CT scans using a support vector regression (SVR) model enhanced by radiomics features.^17^ The radiomics features used include various statistical descriptors from the imaging data, allowing the model to capture intricate details about tissue properties and anatomical features. The study involved 237 patients who underwent brain CT scans. The patients’ data were processed to delineate regions of interest (ROIs) in the brain, and radiomics features were extracted from these ROIs. The benchmark for organ doses was established using fast GPU-accelerated MC simulations, and these doses served as training targets for the SVR model. The SVR model’s performance was evaluated by comparing predicted doses against reference doses with metrics such as the relative root mean squared error, mean absolute percentage error, and coefficient of determination. The results indicated high accuracy and robustness of the model, with the ability to predict organ doses within 1 mGy of the reference doses, showing a maximum absolute error of 1.3% for the brain.

Peng et al developed a CNN based on the U-Net architecture to segment multiple radiosensitive organs from CT images automatically.^18^ Organ dose calculations were performed using the GPU-accelerated MC code ARCHER, designed to be efficient and integrate seamlessly into clinical workflows. The median Dice similarity coefficients for organ segmentation were very high, reflecting accurate segmentation performance (eg, lungs at 0.97, liver at 0.96). The system’s efficiency was highlighted by the rapid processing times, with segmentation taking <5 s per patient and dose calculations under 4 s. Similarly, the technical note by Adamson et al evaluates a V-Net auto-segmentation algorithm designed for paediatric CT scans.^19^ This fully convolutional network (FCN) addresses the challenges of organ segmentation in paediatric CT images, recognizing the considerable variability in anatomy due to different ages and development stages. The study tested the algorithm across various conditions, including differences in CT scanner protocols and patient ages, to assess its performance and generalizability. A dataset of 359 paediatric CT scans with expert annotations was utilized to train and evaluate the model, highlighting the potential of deep learning in improving patient-specific CT dose estimation. The results indicate promising generalizability of the FCN models across different CT scanner protocols and a wide range of paediatric ages, without the need for scanner or age-specific models. The algorithm’s efficacy is further underscored by its application in patient-specific CT dose estimation, where auto-segmented organ contours are used to calculate organ doses. The models achieved mean dose errors within acceptable margins for most organs, demonstrating the utility of FCN auto-segmentation in enhancing the safety and effectiveness of paediatric CT imaging by facilitating precise dose estimation.

A few studies have introduced AI-based methods for real-time, personalized dosimetry in other than CT X-ray imaging modalities. Local diagnostic reference levels (DRLs) can serve as benchmarks for radiation exposure in medical imaging, helping to optimize patient doses.^20^ An article utilizes a neural network to automatically and quickly establish and update local DRLs in interventional radiology, with a focus on endovascular aneurysm repair (EVAR) procedures.^21^ The study aims to develop an automated method to streamline the time-consuming and complex process of setting local DRLs, which are used to optimize radiation protection in interventional radiology. The researchers collected radiation dose reports from 46 consecutive EVAR procedures, which served as the dataset for the development and validation of the proposed system. The method involves using an optical character recognition engine, specifically Tesseract, to automatically extract data from the dose reports such as kerma area product, air kerma, number of exposure images, and fluoroscopy time. The neural network within Tesseract helped in accurately extracting and processing these parameters. The automated process proved to be highly efficient, achieving an accuracy of 99% in recognizing data from the dose reports. It significantly reduced the time required to establish DRLs by 98%, with the new system needing only about 18 s to process data that typically took over 16 min manually.

Tsironi et al introduced an advanced AI-based method for real-time, personalized dosimetry in chest cone-beam CT used in radiotherapy.^22^ This research aims to improve the prediction of organ-specific radiation doses using AI algorithms. The study utilized CT images from 113 patients who underwent thoracic radiotherapy. MC-generated organ dose data were employed to train AI models. AI algorithms were developed and tested for their ability to predict doses to critical organs like the bones, oesophagus, breast, heart, lungs, skin, and overall body. The AI models were evaluated through Bland-Altman plots comparing AI-predicted doses against MC simulation results. The discrepancies in doses were quantified to assess the precision of AI predictions, with mean discrepancies for various organs ranging from 0.5% to 2.5%, and maximum discrepancies from 9.4% to 21.2%. The AI models showed a close agreement with the MC simulations, demonstrating their potential for clinical application.

The current state-of-the-art method for individualized organ dose estimation in CT involves labour-intensive and time-consuming MC simulations. A study investigates the feasibility of using conditional generative adversarial networks for real-time, patient-specific CT organ dose estimation using only CT images.^23^ The authors propose a method leveraging the pix2pix architecture for generating synthetic dose images rapidly. The pix2pix architecture employed for the study features an encoder-decoder generator and a discriminator, which work together to optimize the image translation from CT images to dose images. The study achieved an average organ dose estimation error of 8.3%, with no organ exceeding a 20% error margin. This level of accuracy indicates the method’s potential for clinical application. The feasibility of the method in clinical settings was further demonstrated by developing an automated organ dose calculation pipeline using in-house segmentation tools. This pipeline allowed for the rapid calculation of heart and lung doses per CT slice, taking approximately 2 s per slice.

The adoption of AI in X-ray imaging dosimetry faces several significant challenges, including potential biases in training data and the necessity for robust validation. AI models rely heavily on the quality and diversity of their training datasets. If the data used to train these models is not representative of the entire patient population, it can lead to biased outcomes. Additionally, robust validation is crucial to ensure that AI models generalize well across different patient populations and imaging scenarios. Without thorough validation, there is a risk that AI-driven dosimetry tools might not be reliable when applied to the clinical environment, leading to incorrect dose estimations and potentially compromising patient safety. Addressing these challenges requires a concerted effort to curate diverse and comprehensive training datasets and implement rigorous validation protocols, ensuring that AI models are both unbiased and applicable in clinical practice.

## Conclusion

In conclusion, there is an increasing need for precise, real-time dosimetry in CT imaging due to the high frequency of examinations and the shift towards personalized medicine.

Traditional dosimetry methods, while standardized, often fail to capture patient-specific variables and are generally slow, necessitating a more dynamic approach. Several recent studies provide strong evidence of the benefits of integrating ML techniques into medical imaging, particularly for providing accurate and fast estimates of patient doses with minimal resource requirements. It should be stressed, however, that many of these studies focus on a specific anatomical area, and the models are potentially inaccurate for other body parts or in different institutional settings without retraining or adaptation. Additionally, any uncertainties and biases from the MC simulations, often treated as the ground truth, will also be translated into the AI predictions. While MC simulations remain the gold standard, they too have limitations that must be acknowledged. Addressing these challenges and limitations is essential to ensure the reliability and clinical applicability of AI-driven dosimetry tools.

Integrating ML into routine clinical workflows could revolutionize dosimetry by allowing for real-time, accurate dose assessments that take into account individual patient anatomy. This would be particularly beneficial for specific patient groups, such as those requiring multiple scans, by minimizing cumulative radiation exposure, optimizing imaging protocols, and ensuring consistent dose management. These improvements enhance patient safety while maintaining diagnostic efficacy in clinical settings.

## Funding

None declared.

## Conflicts of interest

None declared.
